# Motor–cognitive interactions in older adults: reliability and comparison of Trail Walking and Stepping Trail Making Tests

**DOI:** 10.1007/s41999-025-01240-w

**Published:** 2025-05-30

**Authors:** Johannes Riis, Steffen Wølke, Mathias Brix Danielsen, Mette Merete Pedersen, Stig Andersen, Martin Grønbech Jørgensen

**Affiliations:** 1 Department of Geriatric Medicine, Aalborg University Hospital, Hobrovej 18-22, 9000 Aalborg, Denmark; 2 Department of Clinical Medicine, Aalborg University, 9000 Aalborg, Denmark; 3 Department of Rehabilitation; Senior and Care Administration, Aalborg Municipality, 9310 Vodskov, Denmark; 4 Department of Clinical Research, Copenhagen University Hospital Hvidovre, 2650 Hvidovre, Denmark; 5 Department of Clinical Medicine, University of Copenhagen, 2100 Copenhagen, Denmark

**Keywords:** Dual task testing, Cognitive–motor interaction, Falls, Cognition

## Abstract

**Purpose:**

Integrated motor–cognitive assessments, such as the Stepping Trail Making Test (S-TMT) and Trail Walking Test (TWT), have shown promise in predicting fall risk and assessing cognitive function. However, their reliability and validity need clarification in European populations.

**Methods:**

This intra-rater test–retest reliability study involved community-dwelling older adults aged 65+. Participants completed both the S-TMT and the TWT across two sessions, 1 week apart, to assess reliability and correlation. The S-TMT required participants to step on numbered squares in sequence on a 1 × 1 meter mat, while the TWT involved walking through a randomized sequence of 15 numbered flags within a 5 × 5 meter area. Test–retest reliability was evaluated using intraclass correlation coefficients (ICC), and correlations were assessed using Pearson correlation.

**Results:**

We included 34 participants (mean age 74.9, 23 female). Both tests demonstrated good-to-excellent reliability: the S-TMT showed an ICC of 0.81 to 0.93, while the TWT showed an ICC of 0.83 to 0.97 based on number of trials. The S-TMT exhibited significant learning effects between sessions, whereas the TWT showed minimal learning effects. A moderate correlation (*r* = 0.48) was found between the tests, indicating they assess different aspects of motor–cognitive function.

**Conclusion:**

The S-TMT and TWT are reliable and complimentary tools for assessing motor–cognitive interactions in older adults. The S-TMT is suitable for single assessment as learning effects limits short-time re-testing, whereas the TWT seems more appropriate for repeated testing. Further studies should validate these results across diverse populations and explore their predictive value for long-term outcomes.

**Supplementary Information:**

The online version contains supplementary material available at 10.1007/s41999-025-01240-w.

## Key summary points

**Aim:**

To evaluate the reliability and the correlation of the Stepping Trail Making Test (S-TMT) and Trail Walking Test (TWT) in a European older adult population.

**Findings:**

Both S-TMT and TWT demonstrated good-to-excellent test-retest reliability. Further, a moderate correlation was found between S-TMT and TWT, suggesting that they evaluate different aspects of motor–cognitive function.

**Message:**

These findings support the integration of both tests into geriatric assessments for better identification of fall risk and cognitive decline.

**Supplementary Information:**

The online version contains supplementary material available at 10.1007/s41999-025-01240-w.

## Introduction

The global society is aging, leading to a significant rise in the incidence of cognitive impairment and dementia [1]. According to the World Health Organization (WHO), approximately 10 million new cases of dementia are diagnosed globally each year, a number expected to double within 20 years [1, 2]. This development underscores the need for early detection of cognitive decline.

Cognitive functions are closely linked to motor functions through shared brain structures and networks, which is clinically evident from that fact that falls, a “failure of motor function”, is a common and serious consequence of cognitive impairment in older adults [3, 4]. Thus, deterioration in these functions often affects both aspects of a person’s ability to function in daily life [5]. This is most pronounced among older adults with limited motor and cognitive reserves, where a minor decline in these functions increases the risk of both falls and dementia [6–9]. Motor and cognitive abilities are usually assessed separately. However, the importance of integrating these assessments to better predict and prevent adverse outcomes such as falls, and cognitive decline was emphasized recently [10–13]

Promising motor–cognitive tests introduced in the last 10–15 years include the Trail Walking Test (TWT) and the more recent Stepping Trail Making Test (S-TMT) [11]. The S-TMT involves stepping on numbered squares in ascending order on a 1x1 meter mat, while the TWT requires participants to walk through a randomized sequence of 15 numbered flags within a 5x5 meter area. The TWT has proven effective in predicting falls in older individuals, while the S-TMT has been highlighted as a practical alternative test with high test–retest reliability in an Asian population, though no studies have yet tested its ability to predict falls [10–12]. With the promise of these tests they should be compared and validated in a European population with description of their reliability before recommending implementation in clinical practice.

This study aims to (1) evaluate the test–retest reliability of both the S-TMT and TWT and (2) assess the correlation between the two tests in a European older adult population. We aim to examine if each test addresses different aspects of motor–cognitive interactions and whether they are reliable tools. We hypothesize that both tests will prove reliable with an intraclass correlation coefficient (ICC) above 0.75, and that the correlation between the results of the two tests is high.

## Methods

### Study design

This study was designed as an intra-rater test–retest reliability study, conducted over two sessions with a 1-week interval between sessions. The study aimed to assess the reliability and correlation of two motor–cognitive dual-task tests: the Stepping Trail Making Test (S-TMT) and the Trail Walking Test (TWT). To minimize potential test order effects, participants were assigned to start with either the S-TMT or TWT based on the parity of their identification number (even: S-TMT; odd: TWT). Identification numbers were assigned sequentially at enrollment without regard to participant characteristics. Each participant maintained the same test sequence across both sessions. While this method balanced test order, it does not constitute full randomization. In addition, participants were tested at approximately the same time of day to avoid circadian rhythm effects [14].

### Participants

Participants were a convenience sample recruited from local activity centers in the Aalborg area, Denmark, in March 2024. Inclusion criteria were community-dwelling older adults aged 65 years or older, with the ability to walk independently with or without walking aids. Exclusion criteria were severe neurological disorders such as Parkinson’s disease or dementia and severe visual impairment. The use of walking aids in day-to-day ambulation was allowed. On the first day of testing, participants filled out a questionnaire on age, gender, medical conditions, falls in the last year, and frailty according to the Tilburg Frailty Indicator (where a score of 5 or more indicates frailty) [15, 16]. The medical conditions included in the questionnaire were hypertension, diabetes, heart disease (including ischemic heart disease, valve disease, and heart failure), chronic obstructive pulmonary disease, and current/previous cancer.

### Testing procedures

**Fig. 1:**
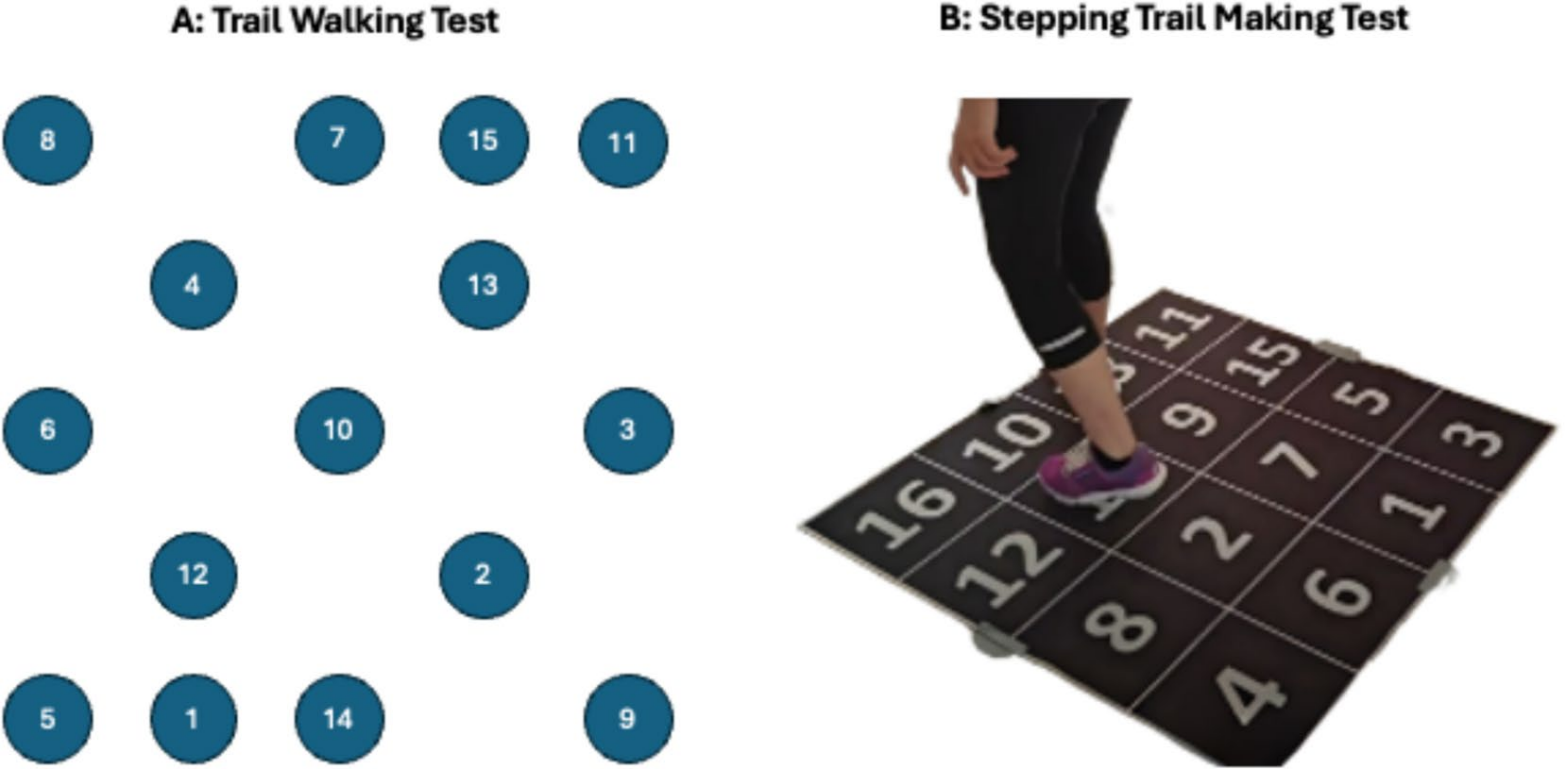
Design of the Trail Walking Test (**A**) and Stepping Trial Making Test (**B**)

Tests were conducted at a local municipal activity center in Aalborg, Denmark in the spring of 2024. Each participant underwent three repetitions (referred to as trials) of both the S-TMT and TWT during each of the two sessions spaced 1 week apart. Each session lasted approximately 15 mins in total. Participants were asked to wear the same shoes on each occasion. If the shoes were inappropriate for the test, participants were asked to perform the tests barefoot. The overall test designs are illustrated in Fig. 1 for the two tests and are described in detail below.

The S-TMT is a motor–cognitive test designed to assess the interaction between cognitive functions and motor abilities in older adults [11]. The test combines elements of traditional Trail Making Tests (TMT) [11–13] with physical stepping tasks to create a more dynamic assessment of a person's ability to process information and execute motor responses simultaneously. The S-TMT is conducted on a square rubber mat measuring 1 meter by 1 meter. The mat contains 16 squares, each measuring 25 cm by 25 cm, arranged in a randomized pattern with uneven numbers on the left side and even numbers on the right side of the mat. Each square is numbered from 1 to 16. The S-TMT requires the participant to use visuospatial skills to locate the next number and maintain balance and coordination while stepping in different directions.

Participants were instructed to step on the numbered squares in ascending order as quickly and accurately as possible. The numbers were initially covered, and once the task began, the mat was uncovered, and the participant stepped on each number in sequence. If a participant stepped on an incorrect number, the test administrator indicated the error, and the participant corrected it, with the time continuing to be measured. The total time taken to complete the task, including any corrections, was recorded in seconds. No upper time limit for test completion was used. Before the actual test, participants were given two practice trials on a separate mat with a different distribution of numbers than the actual test sequence. The practice trials were similar to practice trials in the paper version of the trail making test and were not included in the analysis [11, 17]. See Fig. 1 for an image of the test.

The TWT is likewise a motor–cognitive assessment designed to evaluate an individual’s ability to manage locomotion while visually scanning an area, using their short-term memory and problem-solving ability [10]. Different versions exist [5], however, in the present study, we tested the version where the TWT mimics that of the paper Trail Making Test Part A [10, 17].

The TWT was conducted in an environment where 15 numbered flags were placed in a randomized pattern within a 25-square-meter area (5 × 5 meters). Each flag was surrounded by a 30-centimeter-diameter circle. Participants were instructed to move sequentially from flag number 1 to flag number 15 as quickly and accurately as possible. Participants were required to step into each circle to complete the task successfully. The test was timed using a stopwatch by the same rater to ensure consistency, and the performance was measured in seconds with lower time indicating a better performance. The emphasis was on both speed and accuracy, reflecting the cognitive load required to remember and identify the next flag in the sequence while navigating the course. If participants walked to an incorrect number, they were asked to go back to the previous flag while time continued running. Similar to the S-TWT, participants were allowed two practice trials. Numbers were changed between the practice trial and the actual trials so that the number order was different. The practice trials were not included in the analysis.

### Statistical analysis

For descriptive statistics, categorical variables are given with numbers and percentages, while continuous variables are given as mean and standard deviations (SD) if normally distributed or otherwise as medians with ^1s^t and ^3r^d quarters. Outliers were excluded using the outlier labeling rule where participants with a difference between two tests of more or less than 2.5 SDs were labeled as outliers [18]. Homoscedasticity was assessed by inspection of plots of difference between tests against average of tests.

Learning effects were analyzed by comparing the mean completion times between the first and second sessions using paired *t* tests. Relative and absolute reliability was reported for the first trial, the last trial, and the average of the three trials between the two sessions. Further, relative reliability was assessed using the ICC for each test. Single-measurement two-way random effects ICCs were used for analysis first and third trial at each session and average-measurement two-way random effects ICCs for average of the three trials at each session [19]. An ICC value greater than 0.90 is generally considered to indicate excellent reliability, reflecting a very strong degree of agreement between the repeated measurements. Values between 0.89 and 0.75 indicate good reliability, between 0.50 and 0.74 to indicate moderate reliability, while values below 0.50 suggest poor reliability [20].

Absolute reliability was reported with the coefficient of variation (CV), the minimal detectable change (MDC) and Bland Altman Plots. Coefficients of variation (CVs) were calculated for each participant by dividing the standard deviation of their test and retest scores by their mean test–retest value. The overall CV was then obtained as the average of these individual CVs, expressed as a percentage. A lower CV indicates less variability relative to the mean and therefore greater consistency in the test results. The minimal detectable change (MDC) was calculated using the standard error of measurement (SEM) as an intermediate step. SEM was determined as the standard deviation of the differences between test and retest scores divided by the square root of 2 $\left( {{\text{SEM }} = {\text{ SD}}_{{{\text{test}}{-}{\text{retest}}}} /\sqrt 2 } \right)$. The MDC was then calculated as ${1}.{96 } \times {\text{ SEM }} \times \, \sqrt 2$. The MDC represents the smallest change in a score that can be interpreted as a real difference with 95% confidence, rather than one due to measurement error or natural variability. Bland–Altman plots were reported showing the limits of agreement corrected for learning effects between sessions [21–23].

Correlation between the S-TMT and TWT was evaluated using Pearson correlation coefficients. Average values of the first session were analyzed. In this context, the correlation coefficient (r) of 0.7 and above is considered a high to very high correlation, indicating a robust association between the tests, while correlations of 0.5 to 0.7 were considered moderate, correlation of 0.3 to 0.5 low and correlations below 0.3 considered negligible [24].

No formal a priori power calculation was performed, and a target sample size of ~30 participants was based on what is typical for test–retest studies. However, post hoc sample size calculation confirmed that at least 33 participants were sufficient to find an ICC-value of 0.75 (as aimed) with a 95% confidence interval with a width on 0.3 [25]. All statistical analyses were conducted using R Version 4.3.1.

## Results

### Baseline characteristics

A total of 34 participants were included in the study, with a mean age of 74.9 years (SD = 5.3). Most participants were female (69.7%) and the median BMI was 26.3 kg/m. Regarding health conditions, 1 in 3 of the participants reported having had at least one fall in the previous year, and 3 participants used walking aids. Additionally, nearly half had hypertension, while only one participant was frail according to the Tilburg Frailty Indicator. See Table 1 for full characteristics.

**Table 1:** Baseline characteristics

Age—years, mean (SD)	74.9 (5.3)
Sex—female, *n* (%)	23 (69.7%)
BMI—kg/m^2^, median, Q1–Q3	26.3 (23.9–34.5)
Frailty—TFI ≥ 5	1 (3.0%)
Self-reported falls in last year—Yes, *n* (%)	10 (30.3%)
Use of walking aids—Yes, *n* (%)	3 (9.1%)
Hypertension—Yes, *n* (%)	16 (48.5%)
Heart disease—Yes, *n* (%)	3 (9.1%)
Type II diabetes—Yes, *n* (%)	5 (15.2%)
Cancer/previous cancer—Yes, *n* (%)	7 (21.2%)
COPD—Yes, *n* (%)	2 (6.1%)

*BMI* Body mass index, *TFI* Tilburg frailty indicator, *COPD* Chronic obstructive pulmonary disease

### Learning effect

**Fig. 2:**
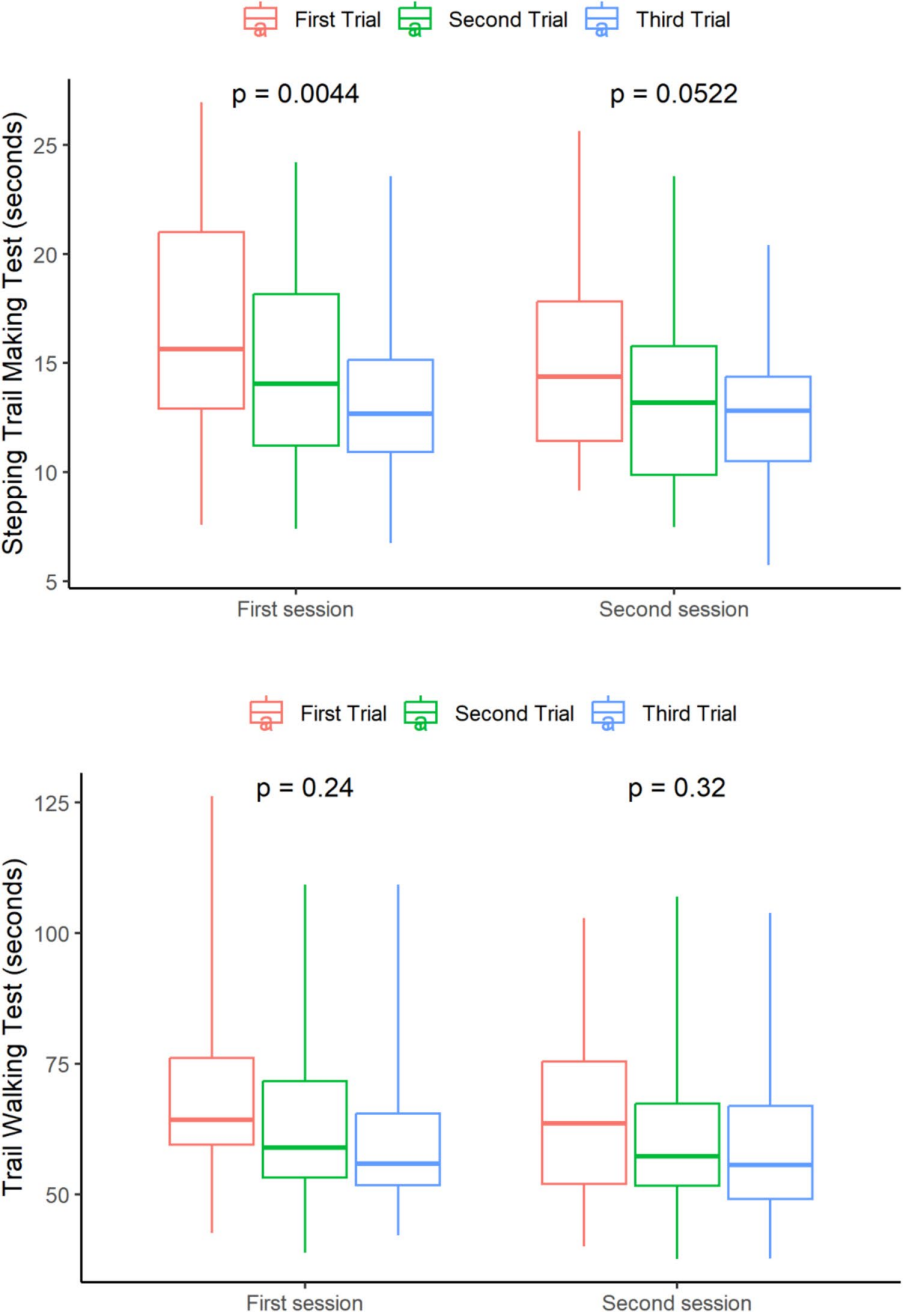
Boxplot of test results by day and trial for Stepping Trail Making Test (upper) and Trail Walking Test (lower)

A significant learning effect was observed in the S-TMT, both within the three trials of each session and between the different session (see Fig. 2). The participants showed improvement in their performance over repeated trials, with the most substantial improvement seen between the first and second trials (mean difference = 1.13 seconds, 95% CI: 0.47 to 1.79). Figure 2 shows that the TWT had a less learning effect, as the participants' performance remained relatively consistent across trials on the same day and with a small difference between test days of 1.85 (95% CI: 0.25 to 3.46) seconds. The mean test scores on the first and third trials, as well as the average for each day, can be found in Table 2, together with the difference between the trials and sessions.

**Table 2:** Reliability measures of the two tests

	Outliers	Results test day 1	Results test day 2	Difference between test days	ICC	CV	MDC (%MDC)
	Number excluded	Mean time (SD)	Mean time (SD)	Mean time (95% CI)	Value (95% CI)	%	Seconds (%)
*Stepping trail making test (in seconds)*							
First trial	0	16.99 (5.27)	15.01 (4.62)	1.97 (1.08 to 2.87)	0.81 (0.42 to 0.92)	11.60	4.90 (30.63)
Third trial	0	13.21 (3.77)	12.72 (3.60)	0.49 (− 0.44 to 1.41)	0.75 (0.55 to 0.87)	10.49	5.12 (39.48)
Average	1	14.78 (4.26)	13.65 (3.76)	1.13 (0.47 to 1.79)	0.93 (0.78 to 0.97)	7.99	3.57 (25.11)
*Trail walking test (in seconds)*							
First trial	0	68.03 (16.81)	65.98 (16.90)	2.05 (− 1.41 to 5.51)	0.83 (0.69 to 0.91)	7.68	19.14 (28.56)
Third trial	1	60.93 (15.22)	58.95 (14.10)	1.97 (− 0.29 to 4.23)	0.90 (0.81 to 0.95)	5.85	12.30 (20.52)
Average	2	62.06 (12.29)	60.21 (12.95)	1.85 (0.25 to 3.46)	0.97 (0.92 to 0.98)	4.36	8.58 (14.04)

*SD* Standard deviation, *CI* Confidence interval, *ICC* Intra-class correlation coefficient, *CV* Coefficient of variance, *MDC* minimal detectable change

### Reliability

The S-TMT demonstrated good-to-excellent reliability, with an ICC of 0.81 (95% CI: 0.42 to 0.92) for the first trial increasing to 0.93 (95% CI: 0.78 to 0.97) when averaging three trials, indicating that the test produces consistent results across multiple repetitions. The TWT also exhibited good-to-excellent reliability, with an ICC of 0.83 (95% CI: 0.69 to 0.91) for the first trail increasing to an ICC of 0.97 (95% CI: 0.92 to 0.98) when averaging three trials. The CV for the S-TMT ranged from 7.99% to 11.60% and the MDC was 3.57 to 5.12 seconds. The CV for the TWT was 4.36%. The MDC for the S-TMT was calculated to be 3.57 seconds. The MDC for the TWT was 8.58 seconds (see Table 2). Bland-Altman plots showing the limits of agreement corrected for learning effects are shown in Supplementary Figs. 1–6.

### Correlation between S-TMT and TWT

**Fig. 3:**
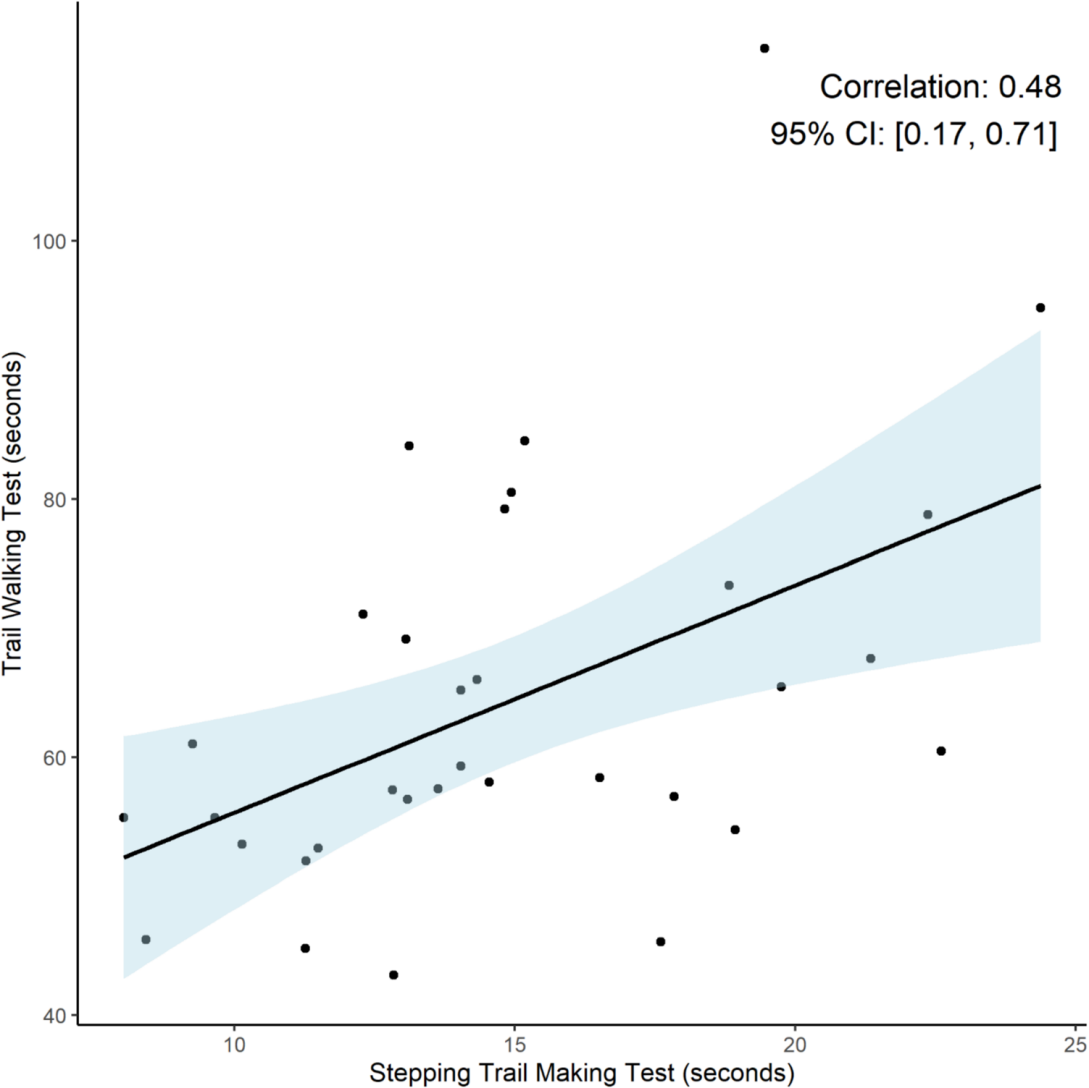
Correlation between Stepping Trail Making Test and Trail Walking Test

We found a low statistically significant correlation between the results of the S-TMT and the TWT of *r* = 0.48 (95% CI: 0.17 to 0.71) (See Fig. 3).

## Discussion

This study provides novel insights into the reliability of the S-TMT and the TWT as tools for assessing motor–cognitive function in older adults from a European country, with additional learning from the correlation between the two tests. Both tests demonstrated good-to-excellent reliability, with the TWT showing higher absolute reliability and less learning effects compared to the S-TMT. Interestingly, the two tests appear to measure different dimensions of motor–cognitive interaction, as indicated by the low correlation between the two tests.

A marked learning effect was observed in the S-TMT, particularly between the first and second trial on the same day. This effect evened out by the third trial, indicating that the participants familiarized themselves with the test and the number sequence. This learning effect is unsurprising as the order of numbers and movement patterns did not differ from day-to-day sessions or between trials within the same day. Learning effects are common in cognitive testing [26, 27] and it is highly likely that the cognitive demands of the S-TMT diminish as participants memorize the number sequence. By the second or third trial, the task changes from engaging cognitive processes to identify the correct sequence into recalling and executing a memorized pattern. The challenge switches to motor execution rather than cognitive decision-making. This may be problematic as one of the merits of the S-TMT is its predictive ability in relation to cognitive decline [12].

In contrast to the S-TMT, the TWT demonstrated less and non-significant learning effects, indicating that the participants' performance was consistent across trials and sessions. This stability makes the TWT particularly useful in situations where repeated testing is necessary, as it minimizes the potential for learning effects to influence the results. It was previously shown that especially the motor component of the TWT is important, and one study showed that only the motor cost and not the cognitive cost of the TWT as a dual-task test was significantly different between older adults with and without cognitive impairment [10, 28]. Considering that learning effects are more common in cognitive testing, and that there are more learning effects on the S-TMT than the TWT it could be speculated that the S-TMT has a higher cognitive demand. This is consistent with better prediction of cognitive decline and lower time to identify numbers due to closer proximity giving higher demands on information processing speed [11], which needs further confirmation.

Both the S-TMT and TWT exhibited good-to-excellent relative reliability. This means that both tests have consistent relative rankings of individual results across multiple repetitions, supporting their use in both clinical practice and in research settings.

The relative reliability of the S-TMT is similar to that found in Osuka et al. (2020), who reported an ICC of 0.82 for the S-TMT in a study conducted in an Asian population [11]. They did not see any learning effects, but they had a much longer time frame between the two test days (six months), suggesting that learning effects are less of a problem with longer time between tests. They also reported an MDC (%MDC) of 10.53 seconds (56.7%), which is larger than our first trial MDC (%MDC) of 4.90 seconds (30.6%) despite similar average completion time of the test. The absolute reliability measures of the S-TMT in our study therefore appear to be better than those reported by Osuka et al. (2020).

In contrast, the TWT's ICC (average over the 3 trials: 0.97) in this study is consistent with the findings of Yamada et al. (2010), who also reported excellent reliability for the TWT in a Japanese sample with an ICC of 0.95 [10]. They did not report measures of absolute reliability, but in our study, the TWT had lower CV’s and %MDC’s than the S-TMT, which further corroborates its high consistency. Overall, the TWT therefore has greater potential as a tool to assess and reevaluate change in performance over time, while the S-TMT with learning effect and worse measures of absolute reliability may be better suited as a screening test.

The moderate correlation between the S-TMT and TWT (*r* = 0.48) is lower than expected based on two motor–cognitive tests supposed to assess the same aspects of functioning. This suggests that these tests assess different aspects of motor–cognitive function but needs to be investigated further. The S-TMT has numbers closer together and therefore may have a lower requirement for visual orientation than the TWT. On the other hand, the S-TMT also takes a shorter time than the TWT, giving less time for orientation and higher demands for processing speed and perhaps working memory. The S-TMT also has no walking component, meaning gait speed does not affect the test. In contrast, the TWT, which requires participants to navigate a path while remembering and locating numbered flags, seems to assess spatial navigation and cognitive-motor coordination more directly, as time during walking can be used for orientation of where to go next, depending on cognitive and locomotor reserve capacity, which can be spared for this task. The tests may therefore by seen as complementary tests addressing different aspects of motor–cognitive interaction, despite both being designed as motor versions of the TMT [29, 30].

The findings from this study suggest that the S-TMT and the TWT can be used together to provide a comprehensive assessment of motor–cognitive function in older adults. Compared to traditional assessments that evaluate motor or cognitive function in isolation, combined assessments may offer a more accurate reflection of real-world challenges faced by older adults, where motor and cognitive tasks often occur simultaneously (e.g., walking while planning or engaging in conversation). This integrated approach may enhance the detection of individuals at risk for functional decline or falls—risks that might be missed when domains are assessed separately [31, 32]. The S-TMT and TWT may therefore provide a valuable addition to existing assessment methods. However, further investigation into the specific aspects of the two tests are warranted.

For practical application, the space required to perform the two tests is worth considering. While being a quick assessment to perform, the TWT demands space (5 × 5 meters) and time to set-up. Therefore, permanent space may be required for the test station. On the other hand, the S-TMT is portable, easy to set up, and can be used in different set-ups and has the potential for use in people’s own homes.

In clinical practice, these tests could be used to identify individuals at risk of falls or cognitive decline, enabling early intervention, however further research is needed to fully explore this potential. Informal feedback suggested that participants found the tests engaging and enjoyable, indicating potentially greater acceptability than traditional cognitive assessments. However, this observation warrants confirmation through formal evaluation in future studies. In research, the potential complementary nature of these tests may offer a robust framework for studying the complex interactions between cognitive and motor functions in aging populations. However, further research into the concurrent validity of the two tests is necessary.

While this study provides valuable insights, there are some limitations to consider. The sample size was relatively small yet was in line with recommendations for reliability studies [25]. Further, the study was conducted in a restricted European population, which may limit the generalizability of the findings to other populations. Also, our sample was relatively healthy, and further research is necessary to corroborate generalizability to more frail older adults or to specific medical conditions. Additionally, longitudinal studies add information on the predictive validity of the S-TMT and the TWT, particularly in relation to long-term outcomes such as the progression of cognitive decline or the occurrence of falls.

## Conclusion

The S-TMT and TWT are reliable tools for assessing motor–cognitive function in older adults. The S-TMT has significant learning effects and may be most suited for single use in assessments and screening, while the TWT has better potential to be used as a repeated assessment of motor–cognitive performance to potential track changes. The potential of S-TMT to assess different aspects of cognitive capacity invites further exploration. 

## Supplementary Information

Below is the link to the electronic supplementary material.

Supplementary file1 (DOCX 1361 kb)
