# Supplementary material for: Motor–cognitive interactions in older adults: reliability and comparison of Trail Walking and Stepping Trail Making Tests
Source: Eur Geriatr Med. 2025 May 30;16(5):1617–25. doi: 10.1007/s41999-025-01240-w (PMC12528192; doi:10.1007/s41999-025-01240-w)
Supplement: Supplementary file 1 — Supplementary file1 (DOCX 1361 kb) [file 41999_2025_1240_MOESM1_ESM.docx]

**Supplementary Figure 1: Bland- Altman Plot comparing the first trial on the Stepping Trail Making Test between test days**

**
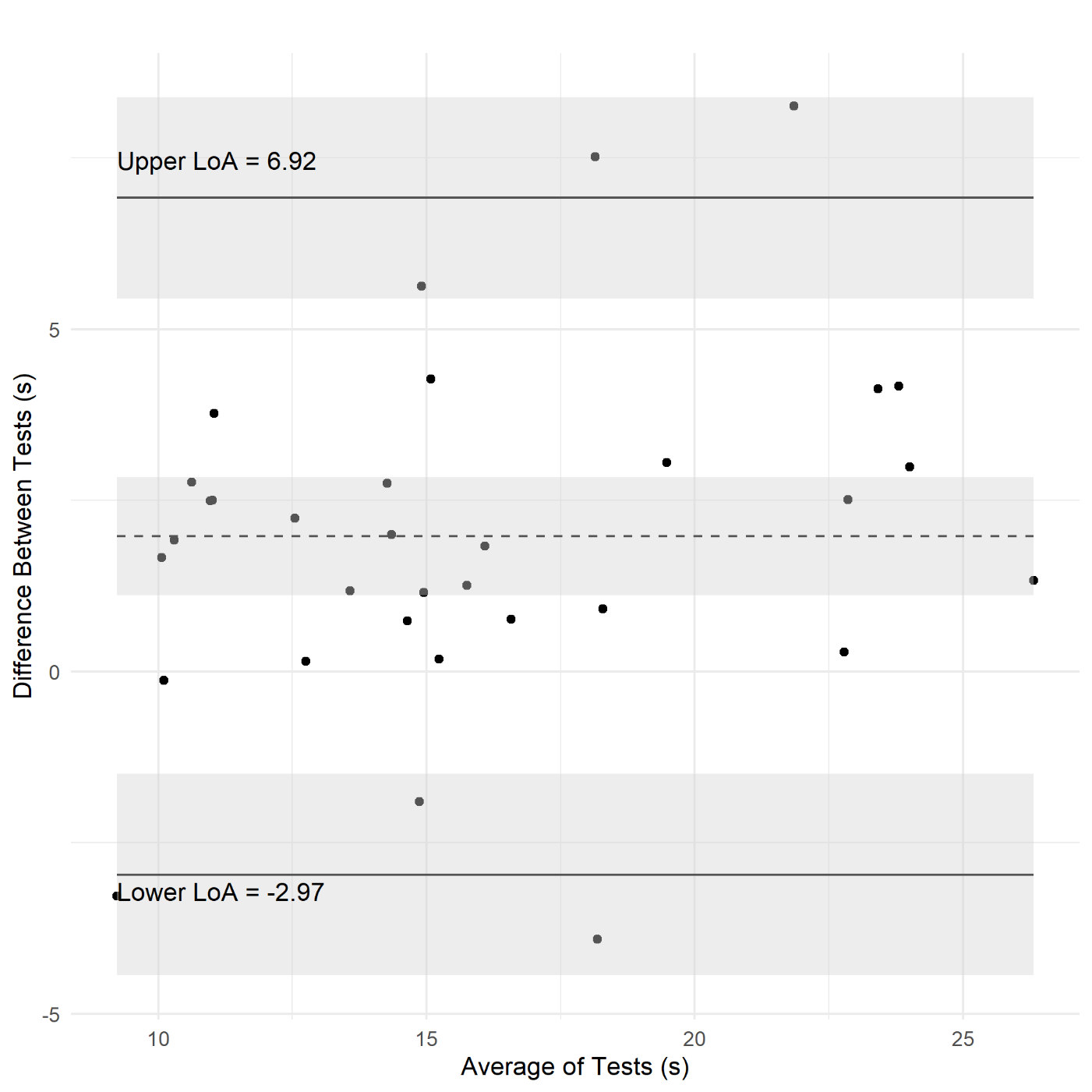
**

**Supplementary Figure 2: Bland- Altman Plot comparing the third trail on the Stepping Trail Making Test between test days**

**
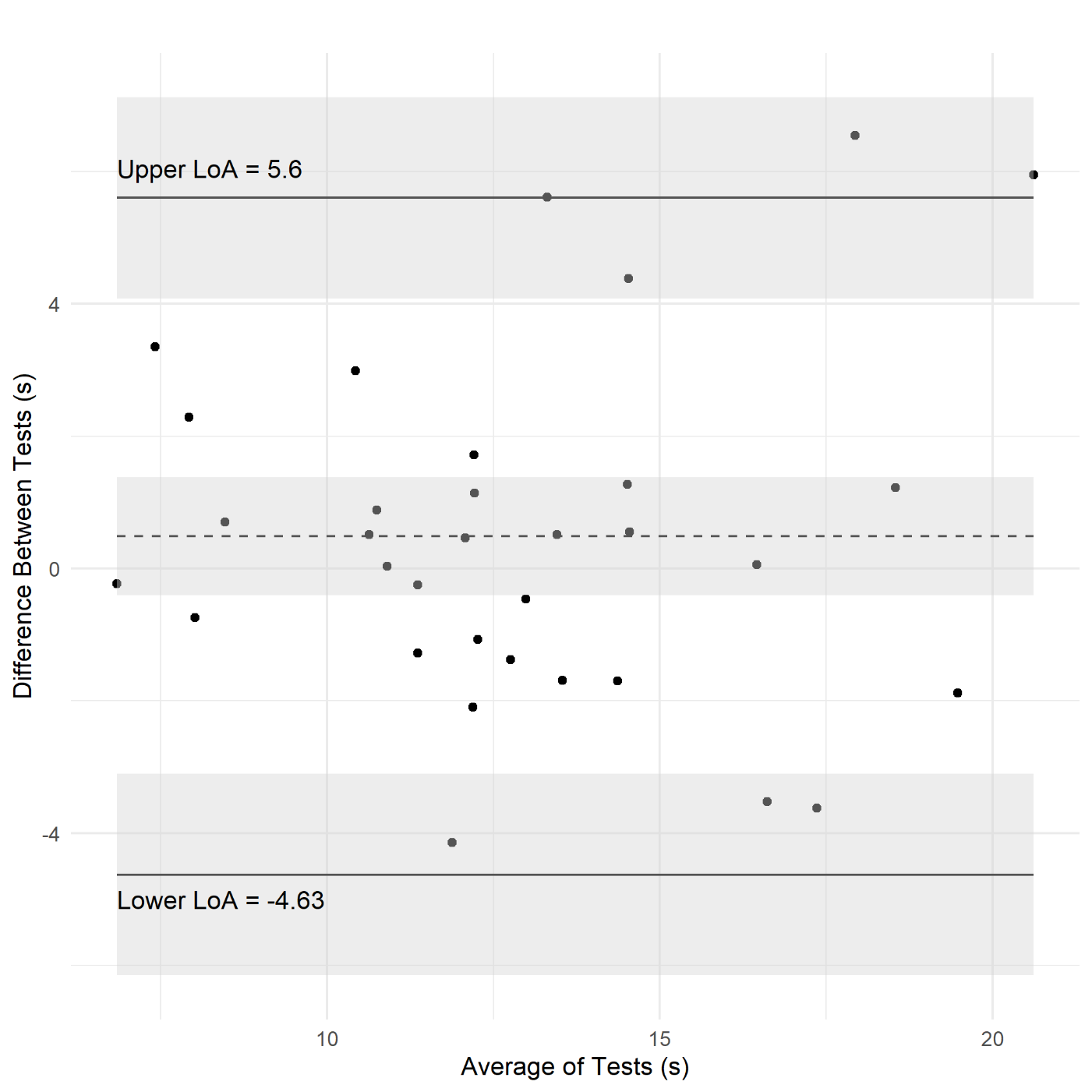
**

**Supplementary Figure 3: Bland- Altman Plot comparing the average across three trails on the Stepping Trail Making Test between test days**

**
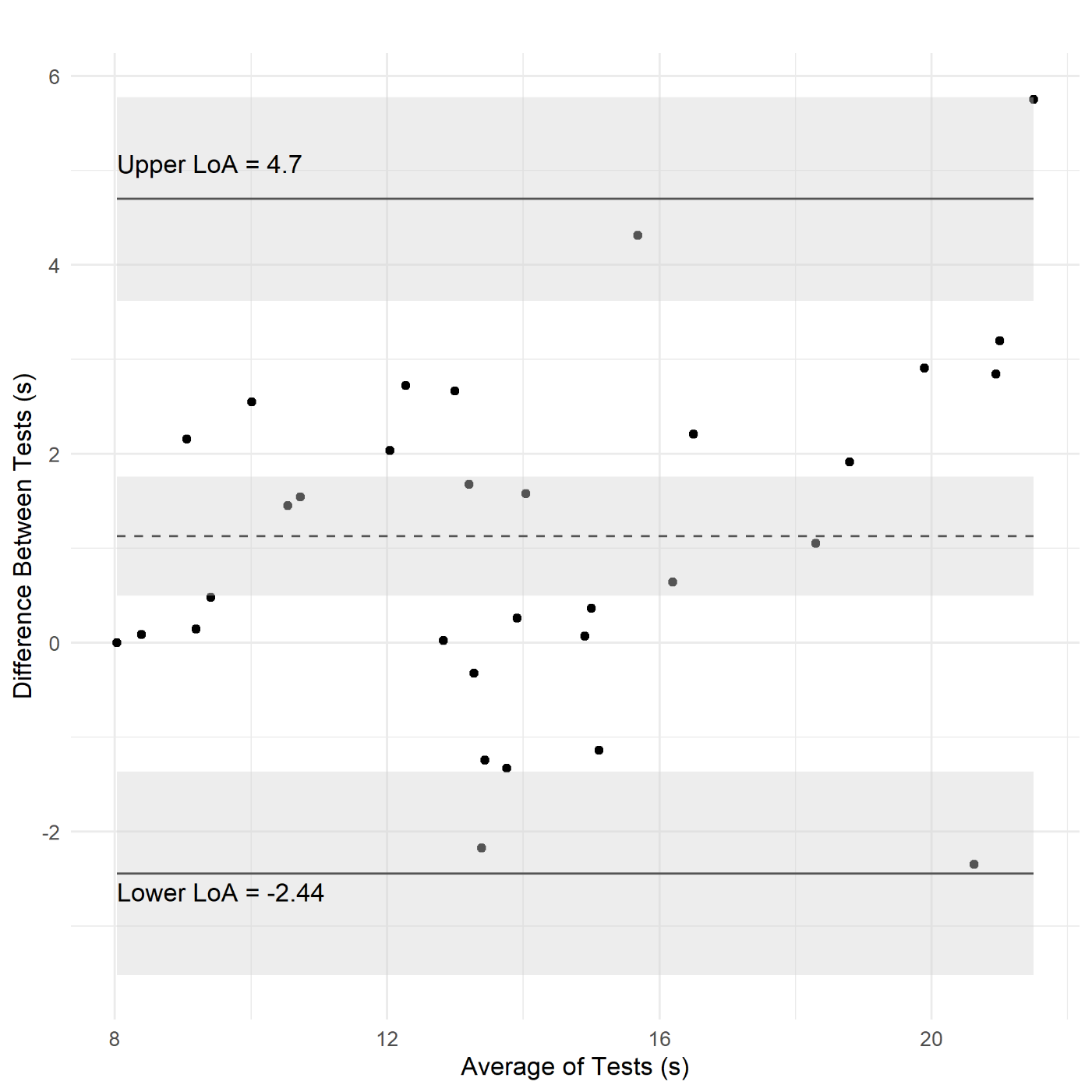
**

**Supplementary Figure 4: Bland- Altman Plot comparing the first trial on the Trail Walking Test between test days**

**
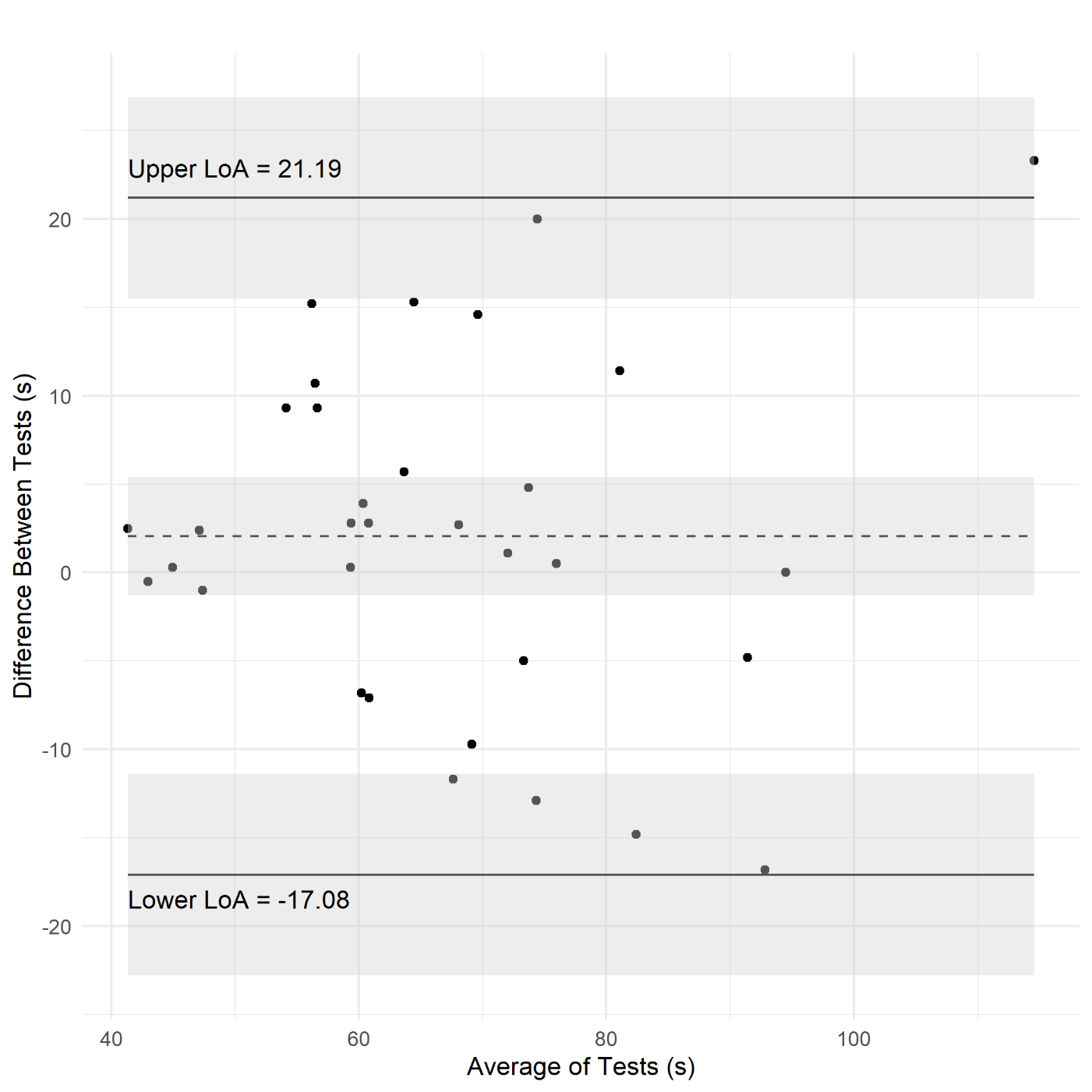
**

**Supplementary Figure 5: Bland- Altman Plot comparing the first trial on the Trail Walking Test between test days**

**
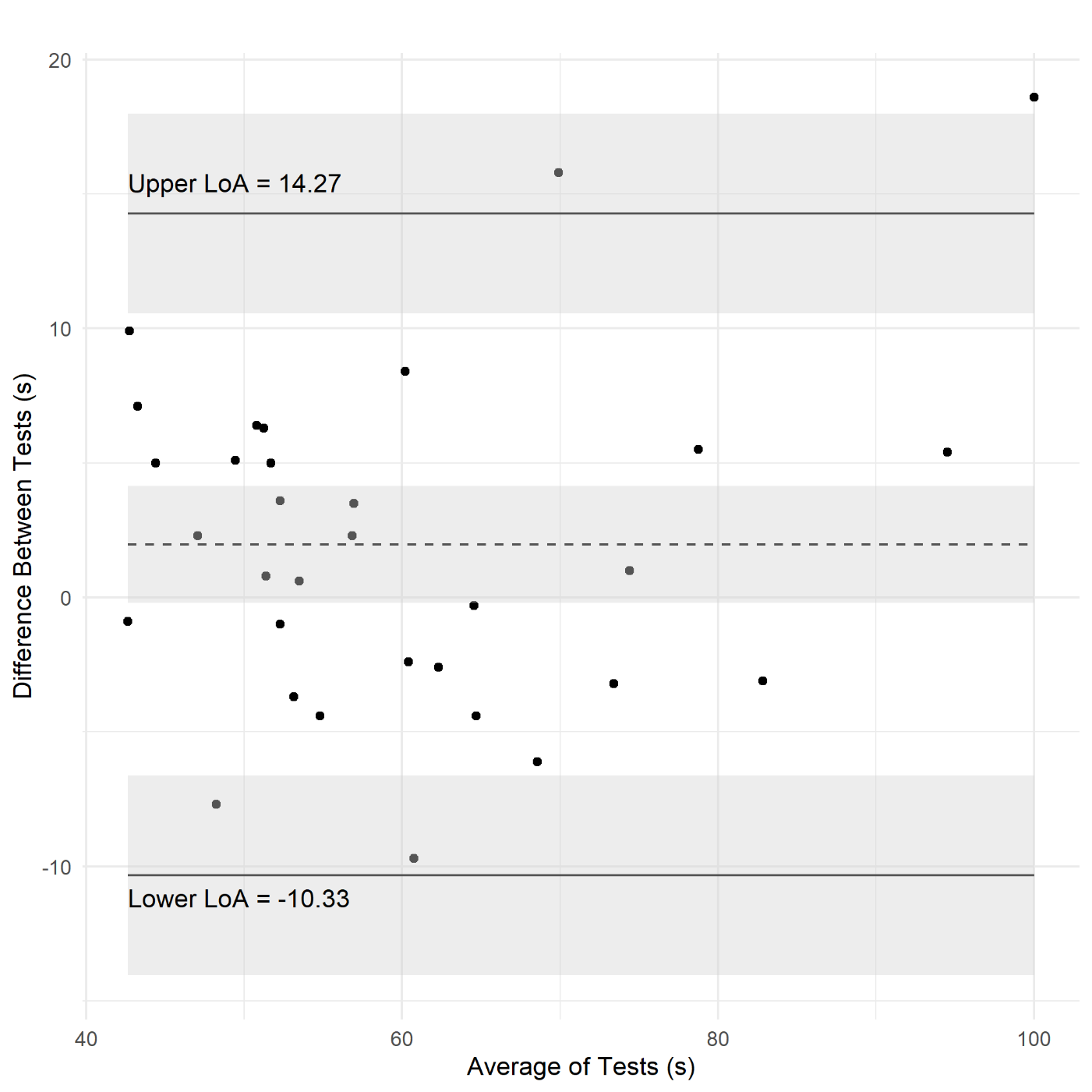
**

**Supplementary Figure 6: Bland- Altman Plot comparing the average of three trials on the Trail Walking Test between test days**

**
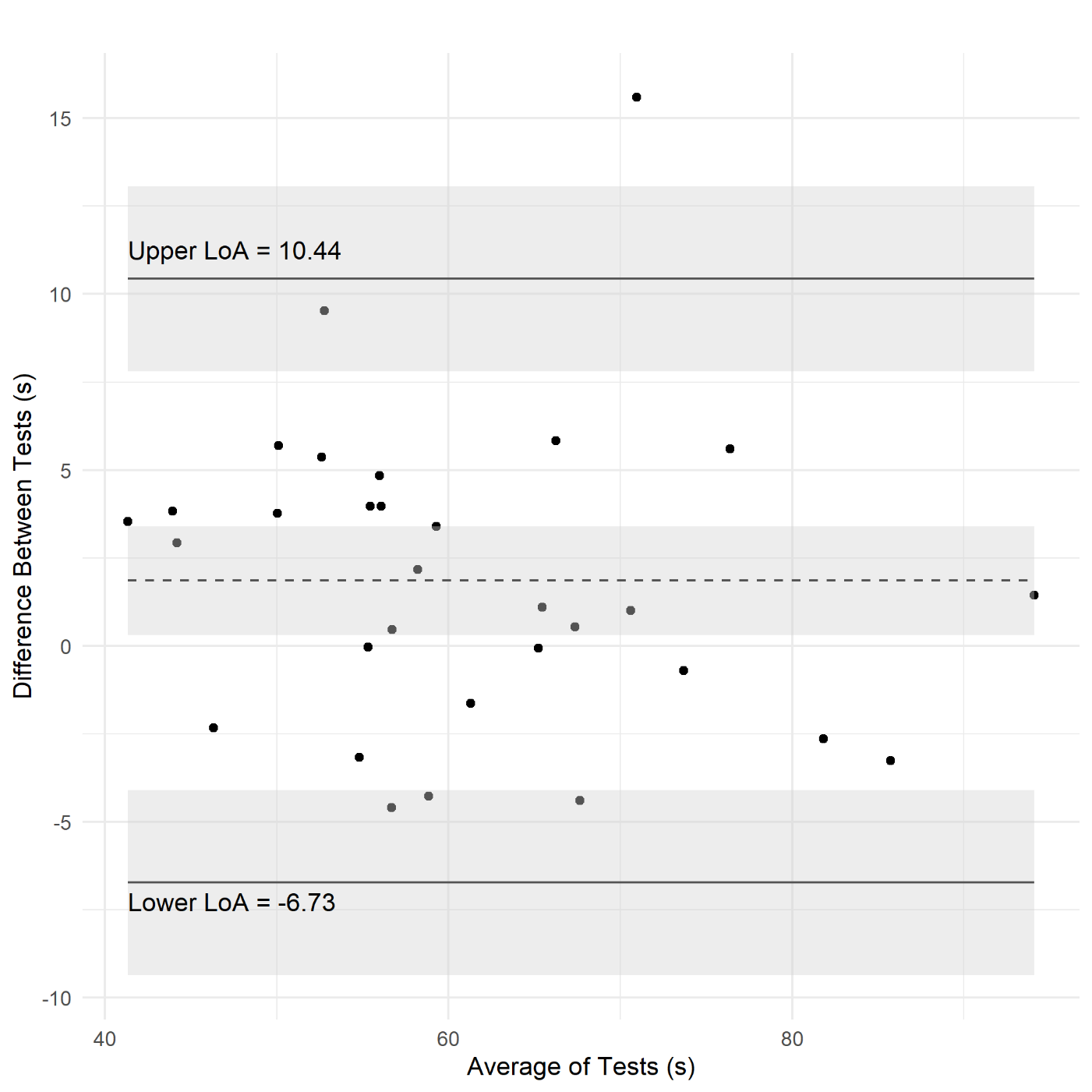
**
